# The Winners and the Losers: Tax Incidence of Gambling in Finland

**DOI:** 10.1007/s10899-019-09899-0

**Published:** 2019-11-03

**Authors:** Tomi Roukka, Anne H. Salonen

**Affiliations:** 1 Economics department, University of Turku, 20014 Turku, Finland; 2 Department of Public Health Solutions, Alcohol, Drugs and Addictions Unit, Finnish Institute for Health and Welfare, Mannerheimintie 166, 00271 Helsinki, Finland; 3 Faculty of Health Sciences, University of Eastern Finland, Yliopistokatu 2, 80100 Joensuu, Finland

**Keywords:** Gambling expenditure, Gambling participation, Tax incidence, Two-part model, Register data, Socio-demographics

## Abstract

Gambling markets have grown rapidly for the last few decades. As a result, gambling is also a very important and common source of tax income for many governments these days. This raises a question about the overall fairness of the gambling taxation systems. In this paper, we aim to study the tax incidence of gambling in Finland. First, we analyse who are the expected payers of the gambling taxes and second, who are expected to be the receivers of the gambling-tax based contributions. In the first part of the study, we analyse the demographic incidence of gambling taxation by using the Finnish gambling 2015 population survey combined with registry based variables. Our data contains 3776 individuals. In the second part of the study, we use data of county level gambling-taxation based contributions to different organisations to analyse how the gambling expenditures are distributed back to citizens in a form of public spending. This study shows that different socio-demographic factors have diverse association with the decisions whether or how much to gamble. The results also suggest that more disadvantaged, i.e. lower income, less educated and rural area living, individuals are expected to be the “losers” of the Finnish gambling taxation system. In other words, the Finnish gambling system is found to be regressive by nature.

## Introduction

In 2016, winning money was the main reason for Finns to gamble (Salonen et al. 2018). According to The Economist magazine, Finland is one of the countries with the highest per capita level of gambling expenditures.^1^ Although, it is widely recognized that gambling does not solely create utility and welfare for the gamblers and society, that is, some people gamble more than they can afford, causing gambling-related harm (Salonen et al. 2018; Browne et al. 2016; Shannon et al. 2017). Usually, this has been the main reason for governments to regulate gambling, i.e. restricting supply to prevent excessive gambling and to minimize gambling-related harm. In fact, the juridical justification for the Finnish gambling monopoly is to reduce and prevent gambling-related financial, health and social harm (Finlex 2011).

Besides for regulation purposes, Finnish gambling revenues have been acknowledged as a base for taxation and government revenue. For decades, the Finnish gambling revenues have been used for financing different organizations from the fields of health, culture and sports, which are perceived as socially beneficial. In addition, gambling taxes (or gambling revenues in case of monopoly supplier) are usually referred to as “painless taxes”, because they are not statutory and gambling is not a necessary good or mandatory consumption (Clotfelter 2005). Furthermore, the earmarking of gambling revenues has frequently been used as a social justification for the existence of the government monopoly and Finnish gambling system where all citizens are advertised being beneficiaries or “winners”. In fact, the gambling revenues have a significant role as a part of public finance in Finland (Kotakorpi et al. 2016).

Despite having a high social status in Finland, the earmarking system of gambling revenues can be seen somewhat problematic overall. A huge drawback of the beneficiary system of the gambling revenues boils down to the mechanism how the gambling revenues are allocated. The revenues are pre-fixed to certain socially and politically accepted purposes. However, a general result in public finance literature (see e.g. Musgrave and Musgrave 1989) is that public expenditures should be allocated as efficiently as possible, irrespective of the source of revenue. Therefore, this kind of rigid earmarking system is not the most efficient way to redistribute the gambling revenues, regardless of the good intentions.

An important question is how the tax-like gambling revenues have been distributed in comparison with the distribution of gambling-tax based contributions to certain predetermined purposes. In other words, what kind of income redistributive effect does the gambling taxation system have in the equity sense? This is particularly important question by the means of political decision making and especially when considering the relative magnitude of gambling in the context of the Finnish economy.

The objective of this study is two-fold: First, to study the demographic incidence of gambling, in other words, how different demographic and socio-economic sub-populations contribute to gambling expenditures. Second, to study how much certain demographic groups are expected to “benefit” from the gambling tax based contributions. Many demographic and socio-economic factors are found to predict gambling participation and expenditures, in addition to the fact that gambling expenditures concentrate on certain individuals (Salonen et al. 2017; Castrén et al. 2018), thus the Finnish gambling system is expected to have some kind of (income) redistributive effects.

The gambling expenditures can straightforwardly be interpreted as the share of paid gambling tax due to flat rate tax, i.e. the tax rate does not depend on socio-demographic variables, e.g. income. However, certain demographic groups may prefer different games with different take out rates. For simplicity, we do not consider how tastes for different games affect the tax incidence of gambling. The analysis is divided between the extensive and intensive decision margins, that is, between decisions whether an individual participates in gambling activity at all, and if individual decides to participate, how much does she decide to spend on gambling. Different socio-economic factors can affect quite differently on these two decisions concerning the consumption of some specific goods, as alcohol, tobacco and gambling. This is because gambling and other so called vice goods might carry some form of a fixed-cost, like a stigma, related to participation. For instance, the consumption of gambling or other vice good may be seen socially blameworthy. Therefore, we also discuss the possible stigma or other fixed-cost associated with gambling participation. Consequently, by combining these two analyses, we seek to uncover the tax incidence of gambling in Finland. In other words, who finances and who benefits from the Finnish gambling system.

### Gambling Expenditures and Demographics

Although people with higher income spend bigger amount of money on gambling, the fact is that lower income individuals have proportionally higher gambling expenditures (Salonen et al. 2017; Canale et al. 2016), implicating that gambling taxes are usually seen as regressive. This concerns especially lottery games and electronic gaming machines (Clotfelter and Cook 1991). Also, men typically spend more money on gambling than women (Scott and Garen 1994; Worthington 2001; Salonen et al. 2018).

Furthermore, low socio-economic status in general, such as low education and unemployment, has been associated with higher gambling participation and expenditure (Scott and Garen 1994; Davidson et al. 2016). Employment status is correlated with disposable income at some level, but it also affects the amount of leisure and even the future insights of an individual. In addition, disadvantaged individuals (e.g. unemployed or on sick leave) usually experience more gambling-related harm and therefore it can be expected that non-working affects both, the participation and spending in gambling (Welte et al. 2017).

In previous studies, marriage has found out to decrease the probability of gambling participation and expenditure (Scott and Garen 1994; Stranahan and Borg 1998; Castrén et al. 2018). The literature provides no clear evidence how retirement status affects gambling participation or expenditure. However, among retired gambling is sometimes used to compensate the increased free-time and declined social interactions (Parke et al. 2018). This predicts higher participation and expenditure relative to working-aged individuals.

Somewhat related to retirement, age is found to increase gambling expenditures in the previous literature (Scott and Garen 1994; Stranahan and Borg 1998; Rude et al. 2014). However, age has found to have a “hump-shaped” effect on gambling, increasing until middle age and decreasing after that (Scott and Garen 1994; Salonen et al. 2018). There is mixed evidence how religiousness affects individuals gambling participation and expenditures and differences between religious groups have been found (Scott and Garen 1994; Rubenstein and Scafidi 2002; Welte et al. 2017). In Nordic countries, living in rural area has shown to contribute positively on gambling expenditures (Rude et al. 2014), but in the studies regarding US the results are somewhat ambiguous (Scott and Garen 1994; Stranahan and Borg 1998; Rubenstein and Scafidi 2002).

### The Allocation and Budgetary Incidence of Gambling Taxes

For the analysis of gambling tax incidence, it is crucial to account how the gambling based revenues, that are earmarked to certain predetermined purposes, are distributed in addition to the distribution of gambling expenditures. For instance, Rubenstein and Scafidi (2002) have examined the distributional effects of gambling in the US, more precisely, what kind of redistributive effect does the Georgian Lottery for Education have. They use household level survey data to estimate the demographic incidence of gambling. For the benefit side, they use county level data of educational attainment, income and race to estimate the distributional effects on lottery-funded programs. In addition, Stranahan and Borg (2004) have studied the budgetary incidence and distributional effects of using lottery tax revenue to finance merit based Florida Bright Futures scholarship. They find the system being regressive, in the way that individuals from lower socio-economic groups tend to pay more as gambling taxes and on the other hand, are less likely to receive scholarships.

All in all, the previous studies which have considered the allocation of the benefits specifically targeted on certain predetermined purposes, indicate that these earmarking systems exacerbate the regressiveness of gambling taxes. That is, low income is associated with higher expenditures, whereas the expected benefits are less than those with higher income. In addition, more educated individuals are expected to contribute less of the gambling tax, but are expected to receive more as contributions.

## Data

### The Finnish Gambling Survey

The individual level cross-sectional data is a combination of the Finnish Gambling 2015 -population survey about gambling, gambling problems, and attitudes and opinions on gambling (Salonen and Raisamo 2015) and the national registry data from the Statistics Finland. Researchers from Finnish Institute for Health and Welfare were responsible for survey design and the survey was conducted by Statistics Finland from 3 March to 8 June 2015. The initial random sample consists of 7297 eligible survey participants from whom total of 4515 interviews was carried out (response rate of 62%). The survey was a Computer Asssisted Telephone Interview (CATI). As the survey was conducted in the first half of 2015, it consists mainly the gambling expenditures from 2014 to beginning of 2015. The registry data contains mostly information from fiscal year 2014, e.g. individual’s disposable income from 2014 is used as an income variable.

The final number of observations included in our analysis is 3776. Most of the individuals were dropped from the dataset because of missing values for some of the explanatory variables (registry based). In addition, 7 individuals were dropped because they had reported frequent weekly gambling, but zero weekly expenditures. These individuals either did not know the actual amount they gamble, did not want to tell the amount they gamble or thought that they were even with the total stakes versus the total wins. One observation was considered as an outlier, having reported weekly amount of gambling about two times the maximum average weekly disposable income of any person. The descriptive statistics in Table 1 show that dropping of observations does not significantly change the central moments of the explanatory variables used in the analysis.

#### Dependent Variables

The dependent variables used in the analysis are survey based; the gambling participation and the average weekly gambling expenditure during the past 12 months. For the gambling participation, a dummy variable was formed indicating whether an individual had reported positive weekly gambling expenditure or not. Participants were asked to approximate their overall weekly gambling expenditure by one question: “Roughly how much money do you spend on gambling in a typical week (EUR)?” (Salonen and Raisamo 2015; Salonen et al. 2017; Castrén et al. 2018). When the respondent hesitated or did not gamble weekly, then he/she was asked to give an estimate of their typical expenditure when participating in gambling. The question may refer to different things regarding different types of games. For example, in lotteries usually the amount is the stakes or the lines bought for the lottery draw, whereas regarding EGMs and casino games the amount may be referred to as the balance between total wins and losses.

#### Demographic Explanatory Variables

Most of the explanatory variables, which are demographic background variables, come from the registries of Statistics Finland. These include continuous variables disposable personal income and age. In addition, dummy variables indicating male gender, being married, belonging to Lutheran church (whether the church tax was paid), unemployment, retirement, the receiving of sick allowances (been on a long sick leave from work) and living in the rural area were formed from the registries. One covariate, whether an individual has completed a university degree, was obtained from the survey, due to a large number of missing registry values.

**Table 1:** Descriptives of the combined Finnish gambling 2015 survey and registry data

	Adjusted sample (3776 obs.)			Original sample (4515 obs.)		
	Mean	Median	SD	Mean	Median	SD
Past year participation	0.73	1	0.45	0.72	1	0.45
Past year expenditures	7.73	3	30.20	18.92	3	744.6
Past year expenditures$^{+}$	10.62	5	34.96	26.28	5	877.41
Disposable income	25,250.61	23,163	30,073.03	24,436.96	22,447	28,533.76
Age	47.14	50	15.75	47.54	50	16.84
Male gender	0.48	0	0.50	0.51	1	0.50
Married	0.51	1	0.50	0.498	0	0.50
Belonging to Lutheran church	0.68	1	0.47	0.67	1	0.47
Unemployed	0.07	0	0.42	0.07	0	0.53
Retired	0.22	0	0.42	0.26	0	0.44
University degree	0.28	0	0.45	0.27	0	0.44
On sickness allowance	0.05	0	0.21	0.04	0	0.19
Rural resident	0.32	0	0.47	0.33	0	0.47

$^{+}$ Conditional on positive expenditures

### Gambling-Tax Based Benefit Data

The gambling benefit data contains specifically targeted gambling-tax based contributions at region level (there are 18 regions) from 2014. The data was collected from the former Finnish legal monopoly gambling companies’ web sites.^2^ The contributions for organizations or purposes labelled as “nationwide” were excluded due to lack of specific location for tracking down the expected receivers or beneficiaries of these contributions. Similarly, the benefits to supporting horse racing culture were omitted due to limited information and traceability. All in all, roughly half of the distributed benefits are labelled as nationwide with no specific location or target group.

Table 2 presents the descriptive statistics of the data of specifically targeted benefits (EUR per capita) at region level that are used in the analysis. In the sample used in the analysis, there are on average 210 individuals per region and individuals “got back” 38.03 euros on average in the form of earmarked gambling revenues at region level. However, it must be noted that the amounts differ highly between regions and purposes.

Despite culture being the largest overall target of the gambling-tax sourced funding, sports is the biggest branch of the contribution purposes that are precisely targeted and can be traced to certain location. Public health also receives large amount of contributions, which however does not show up in the table because public health is defined as broader “nationwide common good”, than those purposes presented in Table 2. For simplicity, these contributions, labelled as nationwide, are treated in the analysis as they would distribute evenly across all demographic groups.

**Table 2:** Descriptives of the gambling-tax based contributions (per capita)

Contribution purpose	Mean	Median	SD	Min	Max
Total	38.03	36.62	10.88	20.36	55.44
Seniors	1.75	1.59	1.69	0	6.07
Children and families	2.29	2.125	1.4	0	5.9
Unemployed	0.48	0.23	0.64	0	2.38
Culture	5.22	3.86	3.66	0.96	12.69
Sports	8.88	8.86	4.56	2.97	18.28
Indiv. per county$^{*}$	250.8	163	262.6	62	1221
Indiv. per county$$^{**}$$	209.78	132	223.73	50	1039

$$^{*}$$Entire sample

$$^{**}$$Adjusted sample

## Analysis

### Analysis of Demographic Incidence of Gambling Expenditures

According to economic theory, the utility from consumption of gambling could, in extreme, be negative due to occurrence of some kind of fixed cost related to the individual’s choice of participation in gambling (Cogan 1981; Moffitt 1983; Scott and Garen 1994). Of course negative expenditures are not possible in real life,^3^ thus these values are censored to zero (non-participation). On the other hand, the survey respondents might not have reported the amount they have gambled truthfully, just telling they haven’t gambled when they actually have or they simply cannot remember correctly if they have gambled or how much, again implying zero observation and possible selection issues. The following distinct feature of gambling expenditure data (large number of zero observations) must be accounted by specific statistical and econometric methods.

There are three widely known and used statistical models that take into account the censoring mechanism of the data, which can be seen as a large probability mass at zero in the distribution function of the dependent variable,^4^ are Tobit model, Two-Part model and Sample selection model. These so called limited dependent variable (LDV) regression models are widely used in the consumption analysis of durable goods and medical expenses, as well as in the labour supply analysis (Cragg 1971; Duan et al. 1983; Cogan 1981). These methods have also been incorporated in the studies regarding the determinants of gambling expenditures (Scott and Garen 1994; Humphreys et al. 2010; Rude et al. 2014).

### Censoring and Corner Solution Models

Censoring is defined as observing always the regressors, **x**, but observing the possible values of latent dependent variable, $y^*$, completely only for a subset of values and incompletely for the rest of the possible values [see e.g. Cameron and Trivedi (2005)]. In the case of censoring from below (or left censoring) at zero, the distribution of *y* can be written as

1 $$\begin{aligned} y = {\left\{ \begin{array}{ll} y^* &{} \text {if }\quad y^* > 0, \\ 0 &{} \text {if }\quad y^* \le 0. \end{array}\right. } \end{aligned}$$

Censoring changes both, the conditional density and mean. The density of *y* is equal to $y^*$ for y > 0, in other words $f(y|x) = f^*(y|x)$ when y > 0. However, when *y* is at the lower bound ($y=0$), the density is a large discrete spike of probability mass that gives the probability of observing $y^{*} \le 0$, i.e. $F^*(0|x)$. Therefore, the conditional density for censoring from below can be written as

2 $$\begin{aligned} f(y|x) = {\left\{ \begin{array}{ll} f^*(y|x) &{} \text {if }\quad y > 0, \\ F^*(0|x) &{} \text {if }\quad y = 0. \end{array}\right. } \end{aligned}$$

Thus, the density can be written as a combination of conditional probability density function (PDF) and cumulative distribution function (CDF) by using an indicator variable defined as

3 $$\begin{aligned} d = {\left\{ \begin{array}{ll} 1 &{} \text {if }\quad y > 0, \\ 0 &{} \text {if }\quad y = 0. \end{array}\right. } \end{aligned}$$

Therefore, the conditional density in the case of censoring from below can be formalized as

4 $$\begin{aligned} f(y|x) = f^{*}(y|x)^{d} F^{*}(0|x)^{1-d}. \end{aligned}$$

Regarding gambling expenditures, however, the problem is not the observability of the dependent variable (gambling expenditures), but rather the fact that many individuals make an optimal decision of non-consuming, i.e. choose a corner solution of not to gamble. Theoretically, the two cases call for the same empirical handling. Although, in the case of corner solution outcomes, the latent dependent variable, $y^{*}$, is just an artificial object, which should not have too much emphasis in our analysis. This is the case when the interest lies on *E*(*y*|*x*) rather than $E(y^{*}|x)$. In our application $y^{*}$ can be seen as “desired” amount of gambling, whereas our interest and usually in other applied empirical work as well, lies on the realized gambling expenditures *y*.

### Tobit Model

The classical estimation approach dealing with corner solution outcomes and censored data is the Tobit model. The most traditional case is when the censoring happens at zero or in other words from below, as in our case. Tobit model assumes that the latent dependent variable is linear in regressors with additive, homoskedastic and normally distributed errors:

5 $$\begin{aligned} y^{*} = x^{'} \beta + \epsilon , \end{aligned}$$

where

6 $$\begin{aligned} \epsilon \sim N(0,\sigma ^2). \end{aligned}$$

The probability that *y* is observed is

7 $$\begin{aligned} F^*(0)&= Pr(y^{*} \ge 0)\nonumber \\&= Pr(x^{'}\beta + \epsilon \ge 0)\nonumber \\&= \varPhi \left( -\frac{x^{'}\beta }{\sigma }\right) \nonumber \\&= 1 - \varPhi \left( \frac{x^{'}\beta }{\sigma }\right) \end{aligned}$$

and $\varPhi$ is the CDF of standard normal distribution *N*(0, 1). The censored density function in the case of Tobit model is then

8 $$\begin{aligned} f(y) = \left[ \frac{1}{ \sqrt{2\pi \sigma ^{2}}} exp {-\frac{1}{2 \sigma ^{2}}(y-x^{'}\beta )^{2}}\right] ^{d}\left[ 1 - \varPhi \left( \frac{x^{'}\beta }{\sigma }\right) \right] ^{1-d}, \end{aligned}$$

where *d* is the indicator variable defined above. The log-likelihood function can thus be written as

9 $$\begin{aligned} ln L_{N}(\beta , \sigma ^2) =&\sum _{i=1}^{N}{d_i\left( -\frac{1}{2}ln2\pi -\frac{1}{2}ln\sigma ^2-\frac{1}{2\sigma ^2}(y_i-x_{i}^{'}\beta \right) ^{2})} \nonumber \\&+(1-d_i)ln\left( 1 - \varPhi \left( \frac{x_{i}^{'}\beta }{\sigma }\right) \right) , \end{aligned}$$

which is a combination of discrete and continuous densities. The Tobit model is estimated with maximum likelihood method. In the Tobit model both decision margins, participation and expenditure, are determined simultaneously and the effects of explanatory variables are similar on both margins.

### Two-Part Model

In most of the empirical applications, however, the Tobit model is too restrictive when stating the same underlying mechanism and parameters for the selection (extensive margin) and the outcome (intensive margin) process. In contrast to Tobit model, Two-Part model (TPM) allows different processes for the censoring (participation, extensive margin) and the outcome (the actual level of gambling expenditures, intensive margin) mechanisms. In addition, if there exists some kind of stigma or fixed cost affecting gambling participation decision, then the Tobit estimation leads to biased estimates and the use of a more general model is needed.

TPM is a generalization of the Tobit model (see Cragg 1971). Formally TPM for the dependent variable *y* can be written as

10 $$\begin{aligned} f(y|x) = {\left\{ \begin{array}{ll} Pr(d=0|x)\quad \text {if }\, y = 0, \\ Pr(d=1|x)f(y|d=1,x) \quad \text {if }\, y > 0. \end{array}\right. } \end{aligned}$$

The participation decision $Pr(d=0|x)$ is usually modelled by estimating Probit or Logit model. For the continuous part of the distribution (positive expenditures), a log-normal distribution is convenient and is usually estimated with ordinary least squares (OLS) regression. TPM can therefore be formalised as

11 $$\begin{aligned}&P(y=0|x) = 1-\varPhi (x^{'}\beta ) \end{aligned}$$

12 $$\begin{aligned}&log(y|x, y>0) \sim N(x^{'}\beta , \sigma ^2), \end{aligned}$$

where the binary participation equation (Eq. 11) is first estimated with Probit by defining dummy variable indicating zero or positive expenditures. Second, the expenditure quation (Eq. 12) is assumed to follow a classic linear regression model, which is estimated with OLS by regressing *log*(*Y*) on a set of explanatory variables *X*.

The previous widely known applications of the Two-Part model include e.g. modelling of health expenditures (see Duan et al. 1983). The estimates of TPM can be compared to those of the Tobit model. If the estimates between these models and therefore the effect of certain variables on the two margins differ, it suggests in our application that there might be some kind of fixed-cost associated with the gambling participation.

#### Sample Selection Model, TPM and Endogenous Selection

The Sample selection model (SSM) (see Heckman 1979), on the other hand, defines a joint distribution for the censoring and the outcome, and then specifies the implied distribution conditional on the outcome observed. Sample selection models are used when the sample is not entirely random. For example when the participation in a survey is voluntary or the quantities asked are determined by the responders themselves, some of the surveyed individuals might be ashamed of their gambling behaviour and refuse to participate at all in the survey or even when participating, might report falsely/remember inconsistently details about their gambling.

SSM is estimated in two separate parts as TPM, but assuming that the error terms from the two equations (selection and outcome) are joint normally distributed. Usually the estimation of SSM is motivated by accounting for endogenous selection, if there are reasons to believe it might be an issue. However, for the identification of SSM, exclusion restrictions are needed. Therefore the estimation of sample selection model is justified, as long as there are convincing instruments for the exclusion restrictions that determine the selection process and can be excluded from the outcome equation for identification of the parameters of the model.

As mentioned above, usually, the use of TPM or SSM is argued to imply a trade off between assumptions about exogenous or endogenous selection. However, TPM is also shown to be a robust estimator in the case of endogenous selection (Drukker 2017). To see this, the observed outcome can be written as a product of participation dummy (*d*) and the value of the variable (*w*), so it either takes value *w* or zero

13 $$\begin{aligned} y = d \cdot w \end{aligned}$$

where

14 $$\begin{aligned} d = {\left\{ \begin{array}{ll} 1, &{} \hbox { if}\ \mathbf {x}\beta + \upsilon > 0 \\ 0, &{} \text {otherwise.} \end{array}\right. } \end{aligned}$$

Now the conditional expectation $E(d \cdot w|x)$ can be written by the law of iterated expectations as

15 $$\begin{aligned} E(d \cdot w|x) =&E_d[E(d \cdot w|x,d)] \nonumber \\ =&E_d[d \cdot E( w|x,d)] \nonumber \\ =&1 \cdot Pr(d=1|x) \cdot E(w|x, d=1) \nonumber \\&+ 0 \cdot Pr(d=0|x) \cdot E(w|x, d=0) \nonumber \\ =&Pr(d=1|x) \cdot E(w|x, d=1). \end{aligned}$$

The both terms, $Pr(d=1|x)$ and $E(w|x, s=1)$ $(= E(y|x, s=1))$, in the right hand side of Eq. 15 can be identified from the observed data. Therefore, $E(d \cdot w|x)$ is also identifiable from data. Consequently, to identify the effect of covariates, $\mathbf {x}$, on gambling expenditures, we do not necessary need to explicitly account for the endogenous selection by estimating SSM. As shown, the TPM estimator is consistent even in the case of endogenous selection. Our main interest in this study are the marginal effects of the demographic variables and thus we can safely ignore the possible endogenous selection issue.

### Estimation Results for the Expected Gambling Expenditures

We start our analysis by estimating a standard Tobit model for a benchmark, which is the classical approach to the censored data. After that, a Two-Part Model (TPM) is estimated to analyse whether the Tobit model for the data is correct and whether there exists some form of stigma or fixed cost associated with some of the demographic factors. Furthermore, the TPM marginal effects are decomposed to analyse more throughout the association between the demographic variables and expected gambling expenditures.

The estimation results of Tobit and TPM in Table 3 reveal that the effects of demographic variables on gambling expenditures vary between these two models. Most of the coefficients have the same signs and significance levels. However, few exceptions exist. According to the TPM estimates, unemployment seems to decrease the probability of participation by 6%, but the effect on the level of expenditures is non-significant (positive coefficient), whereas the Tobit estimate is negative and non-significant. Furthermore, living in the rural area appears to be non-significant in the Tobit model, but on the other hand, has a significant positive effect on the expenditures conditional on participation in the TPM, increasing the expenditures on average by 11.8%.

**Table 3:** Tobit and Two-part estimates for the gambling expenditures

	Tobit model	Two-part model	
		Probit (extensive margin)	OLS (intensive margin)
Dependent variable	Gambling expenditure	Participation dummy	Log (expenditure)
Intercept			− 2.458**
			(1.221)
log(Income)	7.064**	0.177**	0.596**
	(3.360)	(0.007)	(0.270)
Log(Income)^2^	− 0.309*	− 0.008*	− 0.030**
	(0.186)	(0.004)	(0.015)
Male	5.398***	0.147***	0.616***
	(0.630)	(0.015)	(0.045)
Age	0.446**	0.014***	0.027**
	(0.174)	(0.004)	(0.013)
Age^2^	− 0.004**	− 0.0001**	− 0.000
	(0.001)	(0.000)	(0.000)
Married	− 2.924***	− 0.053***	− 0.156***
	(0.672)	(0.016)	(0.048)
Belonging to Lutheran church	1.09	0.055***	0.050
	(0.706)	(0.017)	(0.051)
Unemployed	− 0.947	− 0.060*	0.086
	(1.250)	(0.032)	(0.091)
Retired	− 0.907	− 0.014	− 0.010
	(1.118)	(0.027)	(0.080)
Uni. degree	− 4.393***	− 0.125***	− 0.379***
	(0.754)	(0.019)	(0.055)
Received sickness allowance	2.274	0.057*	0.141
	(1.449)	(0.033)	(0.101)
Rural resident	− 0.072	− 0.006	0.118**
	(0.661)	(0.015)	(0.047)
Observations	3776	3776	2749
Log-likelihood	− 14,409.2	− 2081.2	
Adj. R$^2$			0.11

Estimated marginal effects with heteroskedasticity robust standard errors in parentheses

$$^{*}p<0.1$$; $$^{**}p<0.05$$; $$^{***}p<0.01$$

The coefficient of (logarithmic) disposable income is positive and less than one for the participation equation. In other words, as income increases one percentage the probability of participation increases less than one percentage, 0.177%. In addition, the effect of squared income appears to have negative sign in all equations. That is, the effect of income is positive on participation and on the level of expenditures, but the effect is less the higher the income. Being male has consistently a significant positive coefficient in every equation; being male increases the expected gambling participation and the expenditures by 14.7% and 61.6% respectively. According to both models, age contributes positively on gambling participation and expenditures. TPM estimates suggest that the probability of participation increases by 1.4% with additional year of age, but again with decreasing rate. The TPM marginal effect of age on the expenditures is somewhat larger, 2.7%, and the effect does not fade out as the coefficient of squared age is non-significant.

Marital status has a consistent negative effect on gambling in every equation; individuals that are married participate and spend less on gambling than non-married individuals. In contrast, the results suggest that belonging to Lutheran church contributes positively on both, the gambling participation and the expenditures, although being significant only in participation equation in the TPM. Retirement status does not contribute on either, the gambling participation nor the expenditures according to the estimated models. Those individuals who have completed a university degree have significantly lower levels of gambling participation and the expenditures conditional on participation. Finally, the receiving of sickness allowances (being on sick leave during the last year) contributes positively to the probability of participation and the level of expenditures, however the association is significant only with the participation decision.

In addition, the R-square of the expenditure regression appears to be quite low. This means that we are left with a lot of unexplained variation in the dependent variable, the gambling expenditures. This is the usual case when modelling economic behaviour and decision making; there is a lot of “noise” in the human behaviour. This does not, however, mitigate the relevance of our results and does not imply that the estimated marginal effects are biased. As we are interested in estimating the marginal effects of the demographic variables on gambling expenditures and not trying to forecast or predict the gambling expenditures as precise as possible, we do not need every possible variable that is associated with gambling expenditures. It can actually be more beneficial to leave out additional variables for the analysis of marginal effects to avoid problems as multicollinearity.

#### Decomposition of TPM Marginal Effects

The estimation results between the Tobit model and Two-part model contradict to some extent, which suggests that some of the socio-demographic variables do not contribute to the extensive and intensive decision margins of gambling similarly. Thus, implicating the Tobit model might not be the appropriate model for the data generating process of the gambling expenditures. The TPM marginal effects can be further analysed by calculating the decomposed^5^ effects on both margins and the sum of these two; the total effect of particular variable on the expected gambling expenditures.

By the decomposition of the TPM marginal effects it is also possible to analyse the relative magnitudes of the two components to the total expected gambling expenditures. In addition, as our main interest in this study lies on how different socio-demographic factors contribute on the (total) expected gambling expenditures, it is therefore also crucial to calculate the two decomposed effects. Furthermore, the decomposition is also extremely important because if the mechanism is, for instance, solely through the expenditure margin, it implies that individuals of certain demographic group have higher probability to spend more on gambling conditional on participation. Consequently, this can also be seen as an indicator of increased probability of gambling related problems among particular socio-demographic groups as high gambling expenditures are the most significant predictor of gambling related problems (Markham et al. 2014).

The total effect has two components because the explanatory variables are expected to affect both decisions separately. The (unconditional) expectation of the level of gambling expenditures can be written as

16 $$\begin{aligned} E(G)=Pr(G>0)E(G|G>0), \end{aligned}$$

where $Pr(G>0)$ is the sample proportion of gamblers and

$E(G|G>0)$ is the mean expenditure of those who have gambled. The marginal effect of explanatory variable, $X_i$, on the total expected gambling expenditures is thus

17 $$\begin{aligned} \frac{dE(G)}{dX}=\frac{dPr(G>0)}{dX}E(G|G>0) \nonumber \\ +\frac{dE(G|G>0)}{dX}Pr(G>0). \end{aligned}$$

From Eq. 17 can be seen that the marginal effect of the explanatory variable on expected gambling has two components; the first term is the participation effect (extensive margin) and the second term is the expenditure effect (intensive margin). The proportion of gamblers $Pr(G>0)$ and the mean expenditure of those who have gambled $E(G|G>0)$ are observable from the data. The marginal effects *dE*(*G*) / *d*(*x*) and $dPr(G<0)/dX$ are the estimated coefficients presented in Table 3. Thus, it is quite straightforward to calculate the decomposed effects by using these above described values.

Table 4 presents the decomposed TPM effects and their sum as in Eq. 17. Calculations include only the covariates that had any significant coefficient in Table 3. The sample means of the covariates are used in the calculations. The decomposed TPM marginal effects reveal that many of the variables have vastly different effect on the total expected gambling expenditures and that they clearly differ from the Tobit assumption of the same process for the both margins. However, if the effect of a covariate is different in these margins, it does not necessarily imply that the Tobit model fails to estimate correctly the (weighted) sum of these two effects on the total effect of *X* on expected amount of gambling, *dE*(*G*) / *dX*.

**Table 4:** Decomposed TPM marginal effects

Covariate	Effect		
	Participation (extensive margin)	Expenditure (intensive margin)	Total
Income	0.281	0.434	0.715
Income^2^	− 0.013	− 0.022	− 0.035
Male	0.233	0.448	0.682
Age	0.022	0.020	0.042
Married	− 0.084	− 0.114	− 0.198
Belongs to church	0.087	0.036	0.124
Unemployed	− 0.095	0.063	− 0.033
Uni. degree	− 0.198	− 0.276	− 0.474
Received sickness allowances	0.090	0.103	0.193
Rural resident	− 0.010	0.086	0.076

However, from the Table 4 it can be seen that the decomposed effects of income, male gender, belonging to church, being unemployed, having a university degree and living in rural area are not proportional between the two margins. Furthermore, age, marital status and receiving of sickness benefits all have quite proportional effects. Income increases the expected gambling expenditures proportionally more through the consumption margin. However, the effect on expenditure is declining by a faster rate than the effect on participation. The overall income elasticity (total effect) appears to be less than one. Therefore, the results suggest that lower income individuals have proportionally higher gambling expenditures.

Moreover, men participate and spend more on gambling and the effect on the expected expenditures is mainly via the consumption margin. Keeping other factors constant, the expected gambling expenditures of men are approximately 68 % higher than women’s and two thirds of this effect originates from expected expenditures conditional on participation. One additional year of age increases on average the expected gambling expenditures by 4.2 %. Being married, on the other hand, decreases the expenditures by approximately 20 %. Belonging to the Lutheran church increases proportionally more the expected expenditures via participation margin. Having a university degree cuts the expected expenditures almost in half, the effect emerging a little more through the expenditure margin. Receiving of sickness benefits increases the expected gambling expenditures by 19 % when other demographic factors are kept constant.

As before, regarding being unemployed and living in the rural area, the decomposed effects have opposite signs; negative on the participation probability and positive on expenditures conditional on participation. However, the total effect of these covariates do not have the same signs as the total effect of being unemployed is negative and living in the rural area is positive. Rural residents are expected to have 7.6 % higher gambling expenditures on average when other factors are kept constant and the effect is also clearly more through the expenditure channel.

### Quantile Regression Analysis of Gambling Expenditures

Regarding the consumption of certain vice goods, as gambling, the interest usually lies on the behaviour of individuals that belong to the right tail of gambling expenditure distribution, that is, those who have higher gambling expenditures. Thus, it is important to study how the demographic characteristics contribute to gambling expenditures in different parts of the gambling expenditure distribution (positive part), as this may lead to considerably richer conclusions about the association of certain background variable with gambling expenditures. By estimating the quantile regression model for the positive gambling expenditures, it is possible to study whether there is heterogeneity in how these demographic factors contribute to conditional gambling expenditures. The other advantage of quantile regression, compared to least squares regression, is that it is more robust to outliers and it requires weaker stochastic assumptions for consistency. Consequently, quantile regression, in contrast to OLS, estimates the quantiles of the conditional distribution of gambling expenditures, *y*, given the demographic variables, $\mathbf {x}$. Therefore, quantile regression gives a more overall picture of the data, not just around the mean as OLS regression. Thus, the conditional mean function of least squares estimates can be seen at some level as an incomplete picture of the joint distribution of the response and the explanatory variables in case there is variation in the estimates in different parts of dependent variable’s conditional distribution.

The quantile regression estimator ${\hat{\beta }}_q$ can be written as a minimization problem of objective function (Eq.  18) over $\beta _q$

18 $$\begin{aligned} Q_N(\beta _q) = \sum _{i:y_{i} \ge \mathbf {x'_{i}} \beta }^{N} q|y_i-\mathbf {x'_{i}} \beta + \sum _{i:y_{i} < \mathbf {x'_{i}} \beta }^{N} (1-q)|y_i - \mathbf {x'_{i}} \beta , \end{aligned}$$

where ${\hat{y}}$ is linear in $\mathbf {x}$ and therefore $e = y - \mathbf {x'_{i}} \beta$. From Eq. 18 it can be seen that the estimates of $\beta$ differ between the choices of quantiles, *q*. The special case where $q=\frac{1}{2}$ is the median regression estimator or the least absolute deviations (LAD) estimator.

#### Quantile Regression Results

**Fig. 1:**
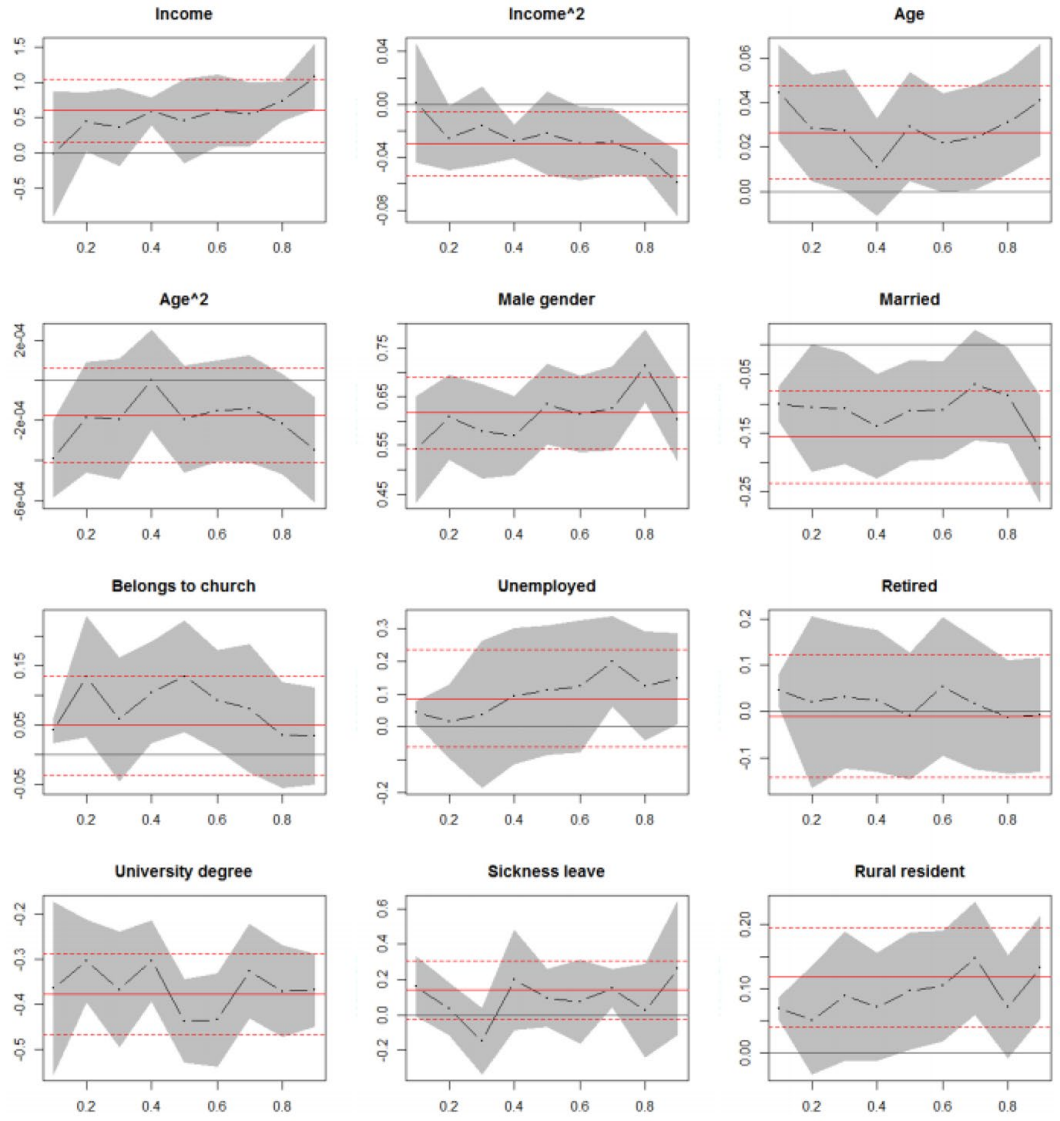
Quantile regression estimates of gambling expenditures

The quantile regression results are presented in Fig. 1, where the values on the X-axis show the quantiles of gambling expenditure distribution and on the Y-axis are the coefficient values. The black dots and lines are the estimated quantile coefficients and the gray shadowed area is the 95% confidence interval of quantile estimates. The constant line is the OLS estimate and the dashed lines are the 95% confidence interval for the OLS estimate.

The results show that most of the variables have quite constant effect over the conditional distribution of gambling expenditures. However, the quantile regression coefficients of income differ statistically significantly from the OLS estimate in the both tails of the conditional expenditure distribution. In the lower tail of conditional gambling expenditure distribution (1st decile), income does not contribute at all to gambling expenditures. In contrast, at the right tail, at the 9th decile, 1 % increase in income is associated with more than 1% increase in gambling expenditures. Although, the effect seems to also dissipate more rapidly in the highest decile. The quantile estimate of male gender differs statistically significantly from the OLS estimate at the 8th decile. However, the estimates below median are less than the average OLS estimate, while in turn being higher above the median.

Moreover, the quantile regression estimates of age do not differ statistically significantly from the OLS estimate. However, Fig. 1 also shows that the quantile estimates of age are higher in the tails of the conditional distribution, so age contributes less on gambling expenditures at the median than in the lowest or highest deciles of conditional distribution. In addition, as was the case with income, the effect of age is also dissipating with faster rate in the both tails of expenditures conditional distribution. The quantile estimates of being married are smaller than the OLS estimate in all, but the highest, 9th, decile. Regarding belonging to Lutheran church, the quantile estimates do not differ from the OLS estimate, suggesting to have quite uniform effect at different levels of gambling expenditure.

Furthermore, the quantile estimates of being unemployed increase along the conditional distribution of gambling expenditures, however non of the quantile estimates differs statistically significantly from the average effect at any point. Having a university degree and being retired have almost constant effect over the whole distribution as the average effect states. The receiving of sickness benefits decreases the expected gambling expenditures and differs statistically significantly from the OLS estimate at the 3th decile. The estimates increase from there on, but not differ from the OLS estimate. Living in rural area contributes less to the gambling expenditures at the lower part of the conditional distribution than at the right tail, however none of the quantile estimates differ statistically significantly from the OLS estimate.

### Analysis of the Distribution of Gambling-Tax Based Contributions

As we have studied how the socio-demographic background factors are associated with gambling expenditures, the next task is to analyse how these compare to the allocation of the gambling-tax based contributions. Thus, we analyse who are the most probable beneficiaries from the public spending of these gambling-based tax revenues. Consequently, by comparing the two estimation results, the demographic incidence gambling and the distribution of gambling-based contributions, inferences about the tax incidence of gambling can be made. In other words, we examine who are the “winners” and who are the “losers” of the gambling taxation system in Finland.

To analyse the distribution of gambling-tax based benefits the following OLS regression is estimated

19 $$\begin{aligned} log(Benefit_{i}) = \alpha + \mathbf {{x'}}_{i}\beta + \epsilon _{i}, \end{aligned}$$

where $Benefit_{i}$ is the level of benefits per capita in individual *i*’s home region. $\alpha$ is constant, $\mathbf {x'_{i}}$ is the vector that contains the set of individual’s background variables as before and $\epsilon _{i}$ is the error term. The estimates of $\beta$ tell how much individuals with certain background characteristics are expected to receive gambling benefits at county level keeping other individual characteristics constant.

Table 5 presents the OLS estimates of the Eq. 19 for the distribution of the contributions sourced from gambling expenditures. The results reveal that on average the expected benefits decrease with income; 1% increase in income is associated with 0.1% decrease in expected benefits. In addition, the effect dissipates as individuals income raises, turning to positive already after relatively small level of income. Thus, individuals with lower income are expected to have proportionally less gambling-tax based contributions at their home region than individuals with higher income.

Furthermore, the results show that individuals that are married, belong to Lutheran church and live in rural area are expected to receive 2.2%, 5.5% and 9.3% less benefits respectively. In contrast, individuals with university degree are expected to receive 3.4% more benefits in their home region on average than individuals with no degree.

**Table 5:** Estimation results for the distribution of gambling-tax based contributions

Dependent variable	Log (benefits per capita)
Intercept	4.095***
	(0.193)
Log(Income)	−0.106**
	(0.044)
Log(Income)^2^	0.007***
	(0.002)
Male	−0.013
	(0.010)
Age	0.003
	(0.003)
Age^2^	−0.000
	(0.000)
Married	−0.022**
	(0.010)
Belonging to Lutheran church	−0.055***
	(0.011)
Unemployed	0.003
	0.019)
Uni. Degree	0.034***
	(0.011)
Received sickness allowance	0.028
	(0.022)
Rural resident	−0.093***
	(0.010)
Obs	3776
Adj. R^2^	0.04

*p < 0.1; **p < 0.05; ***p < 0.01

## Discussion

This article has focused on studying the demographic and tax incidence of gambling in Finland. The results of this study show that variety of socio-demographic factors contribute differently to the decisions whether to gamble at all or, if gambling, how much to gamble. The results suggest that individuals with lower income spent proportionally more on gambling, as the (total) income elasticity of gambling is less than one (inelastic). In addition, the decomposed TPM marginal effects show that income contributes proportionally more on the expected gambling expenditures through the intensive margin. The quantile regression estimates support this notion and suggest that individuals who gamble larger amounts tend to gamble the greater proportion of their income, the income elasticity being above one in the highest decile of gambling expenditure distribution. In contrast, income does not contribute to the spending on gambling at the lowest 10% of the positive expenditure distribution. Thus, income does not seem to play any role in the decision about how much to gamble for those who choose to gamble only a small amount, but on the other hand, those that gamble the most, might gamble excessively relative to their income implying a gambling problem, as Markham et al. (2014) have noted.

Moreover, male gender is consistently associated with the higher probability of participation in gambling and higher level of gambling expenditures. However, the positive association is mainly through the intensive margin. Also, the quantile regression estimates show that male gender contributes more to the gambling expenditures at the higher levels of conditional gambling expenditures. These findings suggest that men are more impulsive gamblers and that they may have more problems to control their gambling and therefore greater tendency of facing gambling related problems.

Married individuals and individuals with a university degree have lower gambling participation probability and lower expenditures conditional on participation, whereas individuals belonging to Lutheran church and those received sickness benefits (been on sick leave) participate more probably and spend more on gambling. Also, all of these previous factors have quite homogeneous effect over the distribution of gambling expenditures according to the quantile regression estimates. In contrast to other socio-demographic factors, retirement status appeared to have no statistically significant association with the gambling participation or the expenditures.

Furthermore, from the TPM estimates and decomposed marginal effects it was seen that being unemployed and living in rural area have opposite effects on the two decision margins, negative on participation and positive on expenditures. This suggests that there is some kind of a fixed cost associated with gambling participation among these socio-demographic groups. In particular, gambling might possess some kind of a stigma among the unemployed, which decreases the probability of participation in gambling whereas, conditional on participation, they gamble on average more than individuals with other employment statuses. The explanation for this might arise from a shame caused by the social outcry that gambling of individuals from certain demographic groups, as unemployed, is not socially-accepted. In addition, living in the rural area may introduce a fixed-cost when participating in gambling because of cost of transportation (including opportunity cost) to the gambling establishment. In rural areas, the gambling opportunities are more spaced out, meaning that it takes more resources to take part in gambling. Both above described mechanisms can lead to the same kind of a reasoning, that is, if individual is willing to accept the cost of participation in gambling, then why not gamble more in that case. This, on the other hand, might also suggest an increase in risk of gambling related problems among the rural residents and the unemployed.

The estimation results regarding the distribution of the gambling-tax based contributions suggested that the expected amount of benefits in individuals’ region decrease proportionally less as individuals’ income increases. Thus, the lower income individuals are expected to receive proportionally less as benefits relative to their income. Combined with the previous results for the demand of gambling, these results together suggest that individuals with lower income and less education, in addition to belonging to Lutheran church and living in rural area, are expected to receive on average less as benefits than they pay as gambling taxes. Consequently, this implies that the earmarking system of gambling revenues in Finland is regressive and the more disadvantaged individuals might the “losers”, whereas individuals with higher socio-economic status are “winners” of the gambling taxation system in Finland. Therefore, the Finnish government regulated and organized gambling system inflicts income inequality to the society as a whole.

The results of this study bring up the question why this kind of an inequality enhancing earmarking system is so widely accepted. Especially as the whole system and gambling supply is government organized. Furthermore, ’the implicit gambling taxes’ are a non-negligible part of the overall tax income of the public sector in Finland. Consequently, the gambling taxes and the earmarking system is expected to have a significant effect on the equity of the overall taxation system by increasing the income inequality in Finland.

### Limitations

Estimating one’s own gambling expenditures and losses can be quite difficult. Gamblers are found to underestimate their losses in the surveys (Williams and Volberg 2009; Braverman et al. 2014; Auer and Griffiths 2017). A relevant point is how the respondent is instructed and how the actual expenditure question is phrased (Blaszczynski et al. 2006). In the survey used here, the total expenditure was asked by one question, instead of separately for different game types, which may cause additional bias. Furthermore, high intensity of play and having a gambling problem increase the probability of reporting biased gambling loss estimates (Braverman et al. 2014; Auer and Griffiths 2017). This is in particular the case regarding e.g. EGMs, which also carry a social stigma and complicate the estimation of expenditures even more. In addition, the layout of the EGMs in Finland is found to be quite disproportional and they are usually placed to living areas with more disadvantaged individuals (Selin et al. 2018), which enhance the fact that gambling expenditures are unequally distributed among different demographic sub-populations. Finally, the self-reported losses correlate with the actual losses from the account registries and the self-reported losses have found to be more accurate with shorter 3-month than longer 12 month time-window like in the survey used here (Auer and Griffiths 2017).

Finally, roughly half of the allocated benefits are labelled as nationwide, i.e. they are contributed to organizations and purposes that are thought as nationwide activity, like organizations promoting general health. Thus, those are left outside this analysis, as those do not have a specific location or sometimes even purpose. We expect that these nationwide contributions distribute quite evenly among different demographic groups, because these are granted for more general public health oriented organisations as Red Cross. However, many of these big organisations operate from the metropolis area, therefore it might amplify the regressiveness of the gambling system. Also, we cannot say that the contributions are surely used for the purpose they are granted for. The improper use of the gambling tax based contributions might rather increase than decrease the regressiveness of the system.

## Data Availability

The Finnish gambling 2015 dataset is available from the Finnish Social Science Data Archive (http://www.fsd.uta.fi/en/). According to a separate contract, the register-based data was available from Statistics Finland (https://www.stat.fi/tup/mikroaineistot/etakaytto_en.html). The data of gambling revenues allocated as contributions is available from the Finnish gambling operator’s website (http://www.veikkaus.fi).

## Footnotes

1 The Economist magazine gives every year an update of the gambling expenditures (or gross gambling revenue, GGR) by country and Finland has long been in the top 5 regarding the per capita gambling expenditures.

2 However, the old three separate gambling operators were merged at the beginning of 2018 to one company, which was named as Veikkaus.

3 Although some individuals could have by definition negative expenditures if the total actual winnings exceed the total actual stakes in a certain period. This matter is however neglected as the data contains only non-negative gambling expenditures.

4 About 27 % of the respondents reported they had zero average gambling expenditures during the past 12 months.

5 See McDonald and Moffitt (1980) for more throughout discussion about the decomposition of LDV marginal effects.
